# Exploring the impact of the COVID-19 pandemic in Euroleague Basketball

**DOI:** 10.3389/fpsyg.2022.979518

**Published:** 2022-09-23

**Authors:** Rûtenis Paulauskas, Mykolas Stumbras, Diogo Coutinho, Bruno Figueira

**Affiliations:** ^1^Educational Research Institute, Education Academy, Vytautas Magnus University, Kaunas, Lithuania; ^2^Research Center in Sports Sciences, Health Sciences and Human Development, CIDESD, CreativeLab Research Community, University of Trás-os-Montes e Alto Douro, Vila Real, Portugal; ^3^University Institute of Maia, ISMAI, Maia, Portugal

**Keywords:** match performance, home advantage, lockdown, attendance, referee decisions

## Abstract

The aim of this study was to understand how training and playing conditions during the COVID-19 pandemic affected the performance of Euroleague Basketball players. Using a non-participant observation analysis, the study compared the seasons before the lockdown (2018–2019 and 2019–2020; pre-pandemic) with the season after restart (2020–2021; pandemic). Paired *t*-tests and Wilcoxon tests were applied for variables with normal and non-normal distributions, respectively. The results revealed significant changes (*p* < 0.05) in several offensive and defensive performance-related variables during pandemic times (without attendance): free throw attempts, free throw percentage, turnovers, three-point attempt rate, fouls (small effect sizes, ESs), points, and possessions (trivial ES). The pre-pandemic HA (70%) significantly decreased after the lockdown, with games played with no crowd (∼51%; *p* = 0.018, large ES). The one-sample *t*-test showed that the HA after the COVID-19 interruption was not significantly greater than 50%, indicating that the HA did not endure during the pandemic condition. Although significant differences between home and away teams were found for most performance-related variables (excepting turnovers) in both pre-pandemic and pandemic conditions, variations of the relative HA were only significant for free throw attempts (large ES), points (medium ES), and turnovers (medium ES). The results of this study showed that performance variables were affected by the COVID-19 lockdown. Thus, these findings may help coaches, players, and referees to counteract unwanted competitive events and improve their overall performance, regardless of the contextual/situational circumstances encountered.

## Introduction

The COVID-19 pandemic posed a particular threat to human health and life, and the spread of its causative agent coronavirus 2 (SARS-CoV-2) was extremely aggressive (Harrison et al., 2020). To protect players from COVID-19, the National Basketball Association (NBA) resumed the competition nearly 5 months later, with the top 22 teams isolated together in a so-called “bubble” (McHill and Chinoy, 2020; Price and Yan, 2021). Other competitions, such as the Euroleague Basketball (EB), decided the competition by six regular championship matches, the playoffs, and the final four competition. In order to limit contagion, some organizations limited the presence of the public and the games were played in specific arenas, allowing only the presence of players and essential staff (Higgs and Stavness, 2021). To restart a new season, a protocol of restrictions was subsequently introduced to maximize the health and safety of players, coaches, and referees (Euroleague, 2021). One of the specific requirements for the competition was physical distancing, regular testing, and adaptation of regional rules in restricting spectators. The experience of playing without spectators is a new and unexplored phenomenon, which created the unique conditions to evaluate the effect of the hosts’ matches (Higgs and Stavness, 2021; Bourdas et al., 2022). The home advantage (HA) phenomenon has been widely explored and is considered a key factor to the game outcome in both individual and team sports, including basketball (Pollard and Gomez, 2013). Previous research established that home teams (63.8%) tend to win more on average, than away teams (50.8%) (Higgs and Stavness, 2021). This was explained by the regional peculiarities of basketball leagues (Gomez and Pollard, 2011), the size of the supporting crowd (Wunderlich et al., 2021), the psychological effect of expectations, and the tactical behavior of the team (Pollard and Gomez, 2013; Price and Yan, 2021). Analysis on the impact of the COVID-19 pandemic on HA in different European professional basketball leagues showed that low-level teams benefited more from playing at their home court (Alonso et al., 2022). Nevill and Holder (1999) proposed that the crowd is able to influence the officials to subconsciously favor the home team. It has also been investigated that in the context of the COVID-19 pandemic, the absence of crowds in soccer has erased HA in the Bundesliga, reduced HA in Bundesliga 2 regarding the performance level, and increased the neutrality of refereeing decisions when giving yellow cards (Link and Anzer, 2022). The EB competition stands out as the strongest basketball league in Europe, and its player game-related perception characteristics are close to those of NBA players (Paulauskas et al., 2018). Studies established that the EB game outcome is influenced by technical statistical variables of box score (Mikolajec et al., 2021) and tactical aspects through the parameters of advanced statistics (Mandic et al., 2019). As such, it is important to understand how crowds can affect this specific context and the final outcome.

Due to the different aims, both offensive and defensive game situations must be quantified to characterize game performance. These game performance parameters may be affected as result of the absence of crowd, which may impact players’ performance, coaches’ strategy, or referees’ decisions (Gonçalves et al., 2021). As far as we know, the effect of empty crowd games on EB is little investigated. In addition, it is unclear how the absence of attendance will affect some performance indicators such as free throws attempts, fouls committed, fouls received, and technical fouls. Thus, a better understanding of the HA may emerge from inspecting the EB players’ performance both before and after the lockdown. Accordingly, utilizing the unique context of the 2019/2020–2020/2021 EB seasons, coaches and analysts might use this evidence to counteract unusual training circumstances, and it will allow officials to perceive and decrease the decision bias created by the home crowd. Therefore, this study aimed to understand how COVID-19 training and playing conditions affected EB players’ in-game performance. Based on previous and current studies investigating the effects of the COVID-19 pandemic on basketball, we hypothesized that (1) unusual season break during lockdown had a detrimental effect on game performance in EB games and (2) the absence of crowd decreased HA in competition.

## Materials and methods

### Subjects

This study used a non-participant observational analysis aiming to compare two different conditions, pre-pandemic in 2018–2019 and 2019–2020 seasons with audience, and the pandemic effect in the 2020–2021 season with no audience. The sample comprised 492 games contested during the pre-pandemic period (16 teams in season 2018–2019 and 18 teams in season 2019–2020), and 306 games played after the lockdown, during pandemic times (18 teams in season 2020–2021). Pandemic matches with a limited or unlimited number of spectators were excluded, namely, all the matches with Russian teams. As this study provides open-access data and does not relate to breaches of confidentiality and the use of personally identifiable information, a special approval for this study from an ethics committee was not required but was performed in accordance with the ethical requirements of the journal.

### Experimental procedure design

Data concerning player profiles and game performance were obtained from the official EB website,^1^ which is consensually considered reliable (Sampaio et al., 2015). The collected variables included match performance (MP) profiles (Table 1) such as points, free throws attempted (FTA), three-point percentage (3P%), two-point percentage (2P%), tree throw percentage (FT%), offensive rebounds (OR), turnovers (TO), blocks, steals, fouls, and technical fouls (TF).

**TABLE 1:** Performance-related variables.

Variables	Operational definitions
Points	total number of points scored by field goals and free throws, Pts = 2 × 2Pt + 3 × 3Pt + FT
Off R	number of points produced by a player per hundred total individual possessions
FTA	the number of free throw attempts
3P%	the percentage of 3-point shots made, 3Pt% = (3Pt/3PtA) × 100
2P%	the percentage of 2-point shots made, 2Pt% = (2Pt/2PtA) × 100
FT%	the percentage of free throws made, FT% = (FT/FTA) × 100
TO	the number of turnovers
FRv	fouls received rate
3PaR	three-pointers attempted
OR	the number of offensive rebounds
Blocks	the number of blocks by a defensive player or team
Steals	the number of steals by a defensive player or team
Fouls	the number of personal fouls committed
TF	the number of personal technical fouls committed
Poss	the number of possessions

Off R, offensive rate; FTA, free throw attempts; 3P%, three-point percentage; 2P%, two-point percentage; FT%, free throw percentage; TO, turnovers; FRv, foul received rate; 3PaR, three-point attempt rate; OR, offensive rebounds; TF, technical fouls; Poss, possessions; Δ, variation; ηp^2^, partial eta squared; *p*, between group-subject effect.

Calculations on a data set representing advanced basketball statistics were also used as performance indicators. To create our data set, we performed a different calculation using the following equations (Oliver, 2004; Kubatko et al., 2007): Offensive rating (Off R) = (points scored/possessions) * 100, Fouls received rate (Frv) = fouls received/possessions, Three-point attempt rate (3PaR) = 3P attempted/field goals attempted, and Possessions (Poss) = plays-OR.

The analysis of MP was developed per match accumulated for both teams comparing Pre-pandemic and Pandemic seasons (Table 2). HA was calculated using HA and Relative Home Advantage (RHA) for both teams comparing Pre-pandemic and Pandemic seasons for each performance variable. The absolute HA was expressed as the number of home wins as a percentage of the total number of games won both at home and away: HA = [(number of home wins/number of home and away wins) * 100] (Pollard and Gomez, 2007). The RHA gives the difference between home and away MP indicators expressed as a percentage of the number of MP indicators performed away: RHA = [(Home MP − Away MP)/Away MP] * 100 (Matos et al., 2020).

**TABLE 2:** Home advantage (HA) in pre-pandemic and pandemic seasons.

Variables	Pre-pandemic		Pandemic		ΔRHA (%)	*p*	ES
					
	HA	RHA (%)	HA	RHA (%)			
Points	51.26 ± 2.06^#^	4.93	50.02 ±1.70^#^	3.90	20.95	0.045*	0.4950
Off R	51.23 ±2.03^#^	4.53	50.00 ±1.73^#^	3.77	16.69	0.530	0.1468
FTA	53.62 ±3.60^#^	14.87	50.06 ±3.63^#^	3.46	76.70	0.002*	0.8171
3P%	50.89 ±2.19^#^	2.97	49.97 ±3.29^#^	3.19	7.54	0.328	0.2307
2P%	50.42 ±2.62^#^	1.71	49.98 ±2.09^#^	0.78	–54.56	0.823	0.0521
FT%	50.42 ±1.77^#^	1.78	49.99 ±1.74^#^	3.23	81.81	0.4643	0.1801
TO	47.84 ±2.79	–7.32	49.96 ±2.74^#^	–7.24	–1.05	0.022*	–0.577
FRv	51.35 ±1.99^#^	4.81	50.05 ±2.15^#^	1.11	–76.97	0.131	0.3633
3PaR	49.76 ±2.22^#^	–1.32	49.94 ±3.46^#^	–1.40	6.53	0.722	–0.081
OR	50.92 ±2.85^#^	4.21	49.90 ±3.49^#^	1.78	–57.67	0.530	0.1468
Blocks	52.71 ±5.04^#^	11.08	49.28 ±10.40^#^	4.18	–62.24	0.184	0.3169
Steals	51.27 ±3.35^#^	3.85	50.02 ±3.64^#^	5.99	55.84	0.334	0.2276
Fouls	48.64 ±2.30^#^	–5.23	49.97 ±2.73^#^	–1.37	–73.84	0.119	–0.376
TF	37.59 ±20.37^#^	–21.30	48.87 ±26.05^#^	–11.46	–46.21	0.384	–0.217
Poss	50.02 ±1.76^#^	0.28	50.00 ±0.44^#^	0.11	–61.84	0.632	–0.135

The values are expressed as mean and standard deviation both in pre-pandemic and pandemic seasons.

Abbreviation: Off R, offensive rate; FTA, free throw attempts; 3P%, three-point percentage; 2P%, two-point percentage; FT%, free throw percentage; TO, turnovers; FRv, foul received rate; 3PaR, three-point attempt rate; OR, offensive rebounds; TF, technical fouls; Poss, possessions; Δ, variation; *p*, between group-subject effect.

^#^Indicates applicable HA differences between home and away teams.

*Indicates significant differences of MP between samples.

### Statistical analysis

Descriptive analysis (means and standard deviation) was performed. All data were assessed for assumptions of normality using the Shapiro–Wilk test. Due to the existence of normal and non-normal data distribution, the differences between MP and HA variables that followed a normal distribution (points, Off R, FTA, 3P%, 2P%, FT%, TO, 3PaR, and Poss for MP indicators; points, Off R, FTA, 3P%, 2P%, FT%, TO, FRv, 3PaR, OR, blocks, steals, fouls, and TF for HA variables) were assessed using paired *t*-tests, while the variables that did not meet normality criteria (FRv, OR, blocks, steals, fouls, and TF for MP indicators, while Poss for HA variables) were analyzed using the Wilcoxon test. The mean differences of HA between the pre- and post-pandemic conditions were analyzed using the Wilcoxon test as the data did not meet normality. In addition, to understand whether the HA existed before and endured after the pandemic, one-sample *t*-tests were applied comparing the observed HA with a null value of 50%, indicating no HA.

Complementarily, effect sizes (ESs) for the variables following a normal distribution were analyzed according to Cohen’s *d* using the following thresholds: small (0.2), medium (0.5), and large (0.8), while for the variables that did not show a normal distribution, the ES was calculated by subtracting the average values and dividing the result by the combined standard deviation converted to the following *r* values: small (0.10), medium (0.30), and (0.50) (large) (Cohen, 1992). The alpha level for all statistical tests was set *a priori* at α = 0.05, and calculations were carried out using SPSS software (IBM Corp., Released 2016. IBM SPSS Statistics for Windows, version 24.0. Armonk, NY: IBM Corp.).

## Results

The results of MP are presented in Table 3. We found differences between the pre-pandemic and pandemic performance variables, describing changes in offensive and defensive behaviors on the court: points (*t* = 1.97, *p* = 0.05, trivial ES), FTA (*w* = 26,947, *p* = <0.001, small ES), FT% (*t* = −4.92, *p* = <0.001, small ES), TO (*t* = −5.56, *p* = <0.001, small ES), 3PaR (*t* = −3.91, *p* = <0.001, small ES), fouls (*w* = 26,308, *p* = 0.009, small ES), and Poss (*t* = 2.47, *p* = 0.014, trivial ES).

**TABLE 3:** Comparison of match performance between pre-pandemic and pandemic seasons.

Variables	Pre-pandemic	Pandemic	Δ	*p*	ES
Points	160.06 ±17.11	157.17 ±15.21	–1.80	0.050*	0.112
Off R	112.46 ±11.02	111.83 ±10.63	–0.56	0.565	0.0329
FTA	17.54 ±4.83	16.22 ±4.64	–7.50	0.001*	0.243
3P%	37.05 ±7.51	37.85 ±7.39	2.15	0.077	0.101
2P%	53.48 ±6.10	53.87 ±5.61	0.73	0.411	–0.047
FT%	76.90 ±8.23	79.49 ±8.09	3.36	0.001*	–0.281
TO	15.38 ±3.14	16.53 ±3.26	7.45	0.001*	–0.317
FRv	28.35 ±3.73	27.94 ±3.92	–1.45	0.088	0.113
3PaR	38.77 ±5.42	39.52 ±5.63	1.94	0.001*	–0.223
OR	30.39 ±6.16	29.74 ±6.50	–2.13	0.332	0.064
Blocks	4.92 ±2.44	4.78 ±2.41	–2.69	0.411	0.0577
Steals	12.82 ±3.57	13.48 ±3.90	5.13	0.061	–0.13
Fouls	40.93 ±5.58	39.84 ±5.62	–2.66	0.009*	0.173
TF	0.61 ±0.92	0.59 ±0.89	–3.64	0.757	0.0258
Poss	71.20 ±3.81	70.35 ±3.51	–1.19	0.014*	0.141

The values are expressed as mean and standard deviation both in pre-pandemic and pandemic seasons.

Off R, offensive rate; FTA, free throw attempts; 3P%, three-point percentage; 2P%, two-point percentage; FT%, free throw percentage; TO, turnovers; FRv, foul received rate; 3PaR, three-point attempt rate; OR, offensive rebounds; TF, technical fouls; Poss, possessions; Δ, variation; *p*, between group-subject effect.

*Indicates significant changes of MP.

**FIGURE 1:**
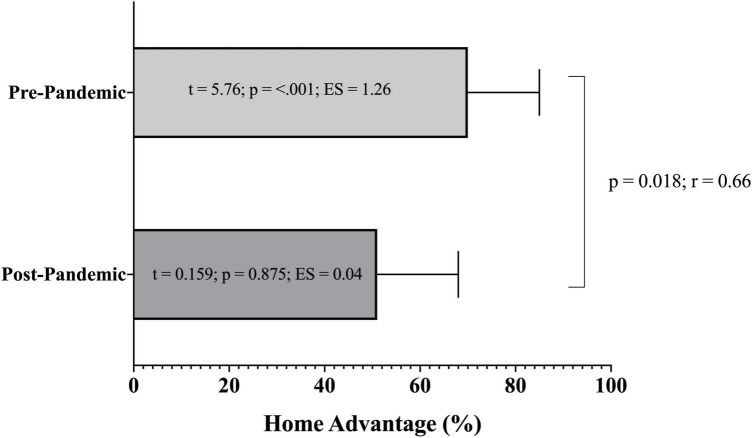
Analysis of home advantage (HA) according to the pre-pandemic and pandemic conditions.

Figure 1 illustrates the HA in the change of match outcomes in pre-pandemic (regular attendance) and pandemic (absence of crowd) seasons, showing significant differences between samples pre-pandemic (HA = 70%) and pandemic (HA = ∼51%; *W* = 142, *p* = 0.018; large ES), suggesting a decrease in the HA under the pandemic condition. When analyzing the HA with a null value of 50%, results revealed a significant difference for the pre-pandemic condition (*t* = 5.76, *p* < 0.001, large ES), yet the difference was non-significant for the pandemic condition (*t* = 0.159, *p* = 0.875, trivial ES), suggesting that the HA existed before the lockdown, but it disappeared after the return to play.

Relative HA values for each performance indicator are presented in Table 2. Overall, there were statistical differences between home and away teams in pre-pandemic and pandemic conditions for all performance-related variables (*p* < 0.001), except TO in the pre-pandemic condition; however, variations of the relative HA were only significant for free throw attempts (*t* = 3.562, *p* = 0.002; large ES), points (*t* = 2.157, *p* = 0.045; medium ES), and turnovers (*t* = −2.517, *p* = 0.022; medium ES).

## Discussion

This study aimed to understand how the COVID-19 lockdown affected EB players’ in-game performance and HA. The main results revealed that the COVID-19 pandemic had a twofold effect: (1) an unusual break with its own duration and limitations before season affected MP in the pandemic EB season, and (2) EB games without crowd established a significant shift in the distribution of match outcome, annulling the HA in the tournament.

### Performance change during the pandemic

This study shows that the average number of points, possessions, fouls, and the number of FTA scored by all teams decreased, while the number of steals, TO, 3PT%, and FT% increased during the pandemic season compared to the pre-pandemic season. Previous research has shown that pandemic confinement has a composite effect on an athlete’s detraining (Bisciotti et al., 2020). This can be attributed to partial or complete loss of previous physiological adaptation to physical exertion (Rodriguez-Fernandez et al., 2018), changes of body mass and composition, a loss of efficiency of neuromuscular and cardiovascular systems, and, consequently, a loss in strength, speed, flexibility, and endurance and an increase of the risk of injury (Bosquet et al., 2013). Sousa et al. (2019) argued that a few weeks of inactivity or lower level activity are sufficient for the decline of physiological capabilities, unless specific programs of training are carried out. In general, interruptions in the training process due to injuries, or other factors, are an ordinary occurrence for single athletes. However, 41 weeks without an official competition for high-performance basketball teams is an unprecedented case in sports practice. Physical fitness seems to be important factor for the decrease in points and FTA during the pandemic season. Despite the small effect size, these two variables have been shown to be important for match outcome and team quality (Kozar et al., 1994; Lorenzo et al., 2010), highlighting the importance that coaches may give to its training. Similarly, in the training environment, coaches apply several boundary conditions to promote the emergence of collective behavior and coordination patterns, considering players’ interaction with teammates and opponents, manipulating technical and tactical-related variables (Ric et al., 2016). Considering that technical performance and tactical behavior are learned and refined not only in competition but also in a training environment, it is possible that the interruption may have contributed to the loss of some collective behavioral trends, leading to the decrease in some performance variables during the pandemic.

Previous studies noted a combination of technical, tactical abilities, and a high degree of physical fitness on optimal performance in basketball (Ziv and Lidor, 2009; Mancha-Triguero et al., 2019). On the other hand, available reports have shown that following COVID, players may require more time to recover from both training sessions and matches (Bourdas et al., 2021). Under fatigue, players are likely to adopt more individual and 1 vs. 1 situations and less collective behaviors (Kunrath et al., 2020), which may help justify the increase in TO in the pandemic. In addition, players’ decision-making results from their ability to perceive the position of teammates, opponents, the ball position, and the target location (Mateus et al., 2019). However, the ability to perceive the relevant information from the environment seems to be affected under periods of mental fatigue, which seems to emerge more often following the COVID lockdown (Lorenzo Calvo et al., 2021), contributing to an increase in the number of TO (Cao et al., 2022). On the other hand, previous research has shown that home crowds increase the chance of winning home games, supported by psychological factors such as team identity or territorial defense mechanisms (Alonso et al., 2022). In this study, fouls presented lower values in the pandemic. Despite available data with the total number of several performance indicators, a better ecological understanding of how behaviors flow between teams during performance is needed. Thus, the results can be explained both by the behavior of the players’ roles in the team strategy and by the referee’s bias and should be treated with caution. However, and based on previous evidence (Wunderlich et al., 2021), referee bias appears to be the most viable explanation for teams being sanctioned more in the presence of spectators.

There is evidence that quality of offensive plays allowed a better process of perception, decision-making, execution, and best performance by players (Gomez et al., 2008). In fact, cognitive factors including past experience, motivation, and development largely contribute to this process (Wu et al., 2013). Therefore, the results of performance such as 3P%, 2P%, and FRv rates showed no such differences between pre-pandemic and pandemic seasons, suggesting similarity in these technical and tactical variables.

The lack of seasonal rhythm, unfavorable periodization, or psychological stress can be attributed to a change in tactics, or physical or technical abilities. A significant difference in performance may also be related with environmental factors (empty arenas) and a change in referee bias (due to the absence of home crowd) (Link and Anzer, 2022). Since this observational study cannot conclusively clarify the underlying reasons, causes, or mechanisms of performance change between time periods, our suggestions may be speculative to some extent.

### Impact of home advantage on match performance

While the HA during the pre-pandemic condition was set at ∼70%, this value decreased for ∼51% during the pandemic condition. These results suggest that the absence of crowd influences the HA, contributing to its reduction. Similar findings have been found by a study comparing the COVID-19 effect in the HA in five European basketball leagues (i.e., Spanish Liga ACB, German Bundesliga, Italian Lega Basket Serie A, A1 Ethniki Greek League and the Israeli Basketball Super League) (Alonso et al., 2022). The authors have found a decrease in the HA, and these results were consistent independently of the team ability level (i.e., low, medium, or high). The crowd support has been found as an important and contributor factor for home team performance (Leota et al., 2021; Ehrlich and Potter, 2022). Despite that, available literature on the impact of the number of spectators, occupancy rate, and noise levels on HA has yielded controversial results. While some studies showed that the HA did not completely disappear in empty stadiums (Wunderlich et al., 2021), other research on the influence of absolute spectator numbers (Moore and Brylinsky, 1993), stadium occupancy (Agnew and Carron, 1994), or noise levels (Salminen, 1993) have shown that spectators do not directly (or only to a very limited extent) take effect on the HA. More recently, studies showed that in certain leagues, the HA totally disappeared in home games without spectators (Fischer and Haucap, 2020), while others presented a lesser HA magnitude (Sors et al., 2022). However, when considering the basketball game, the crowd support seems to have an important effect on team performance. For example, previous reports have shown the importance of crowd support in the effort developed by the players to win rebounds (Leota et al., 2021). Offensive rebounding has been shown to be an important performance indicator for team success (Csataljay et al., 2012). In this study, the teams performed less OR after the pandemic situation than in the pre-pandemic situation. The lower number of offensive R may have contributed to the lower number of points during the pandemic situation. In addition, the absence of crowd also seems to negatively affect FTA and blocks.

The results from this study seem to suggest that indicators such as FRv rate, Off R, blocks, fouls, and TF showed small decreases (i.e., ES values) during the EB pandemic season. Fouls and TF are infractions of the rules penalized and interpreted by referees. In EB matches with spectators, home teams received less fouls and TF (disciplinary sanction) than in matches without audience. Also, in the presence of crowd, home teams were able to execute more offensive actions than visiting teams. These results about disciplinary sanctions are in line with previous studies on soccer (Link and Anzer, 2022). The difference in disciplinary sanctions disappears or is even slightly reversed when the crowd is absent, which supports the idea that the spectator presence is likely to be the only or predominant reason for biased referee behavior (Wunderlich et al., 2021). More equal treatment of fouls when played in the absence of spectators could lead to a loss of HA in blocks and off rebounds. During shot blocking, the defender is not allowed to make contact with the offensive player’s hand, and during the offensive rebound, contact tolerance limits are set by referees, who decide to indicate a foul or not. An experimental study proved that referees use crowd noise as a cue to evaluate the severity of fouls (Unkelbach and Memmert, 2010).

Apart from the fouls and TF, the crowd also seems to impact the team rebound behavior. In this study, we found a decrease in the OR from the pre-pandemic to pandemic season. A previous report also showed a decrease in the number of rebounds when playing without fans compared to those in the games with spectators as it seems that the crowd amplifies players’ effort to rebound the basketball (Leota et al., 2021).

In addition, this study showed that in the absence of spectators, home teams increased the FT% compared to away teams. The large effect found in this variable and its importance on the final match outcome (Csataljay et al., 2009) suggests that it is an important factor to be considered when analyzing HA. Waters and Lovelll (2002) found that psychological effect of expectation is based on the idea that players, coaches, fans, referees, and media are well aware of the phenomenon of the HA, and different expectations of the players at home and away matches could have a self-enforcing (increased HA) or self-locking (decreased HA) effect. The improved FT% in home games without spectators supports the claim that the players have significantly higher perceptions of the team’s confidence at home games (Waters and Lovelll, 2002). At the same time, the probability remains that away teams may have been less affected by external pressure in this context and then performed better than home teams (Tilp and Thaller, 2020).

During the pandemic season, the HA has significantly decreased, resulting in an increased relative HA in points (medium effect) and FTA (large effect) and an increase in TO (medium effect). According to available research (Figueira et al., 2022), free throws and turnovers were considered the most discriminatory variables between winning and losing teams. In the game, complex collective behaviors emerge as dynamic space–time interactions between players unfold (McGarry, 2009). Thus, it is difficult to attribute a cause–effect relationship between the presence of an audience or not. However, previous reports (Pojskić et al., 2011) have shown that under attendance pressure, home players are more careful, which may indicate a greater tendency to take more risks in the absence of crowds. On the other hand, the fact that home teams with higher free throw levels have a larger HA (Harris and Roebber, 2019) seems to support the present results.

Overall, this study provided important practical applications for coaches. Thus, understanding the influence of attendance on the final result of the game can help coaches formulate strategies to improve the overall performance, regardless of the contextual/situational circumstances encountered, such as the training of cognitive-emotional regulation during home and away games, the game strategy (substitutions and specific time-outs), or the stimulation of players’ territoriality. Nevertheless, some limitations may be acknowledged. For example, this study has only addressed the HA effect, while players’ performance is also affected by other contextual variables, such as match status, game period, or quality of the opposition that may impact the presented results. Thus, future studies should use multivariate statistical treatment, which allows controlling the effect of important variables such as team ability, physical conditioning data, or stadium occupancy rate. In addition, apart from the technical performance, a more holistic understanding of the changes of players’ performance may be achieved when analyzing the players’ tactical and physical performance. Lastly, most of the studies inspecting the effect of the COVID lockdown has been performed in male athletes, and thus, future studies should also analyze female competitions.

### Conclusion

The COVID-19 pandemic has affected the basketball EB games in terms of MP and HA. We found that most performance results had significantly diminished after restarting the competition. The lack of seasonal rhythm, unfavorable periodization, or psychological stress could be attributed to a change in tactics, or physical or technical abilities. In addition, several performance-related variables changed in games played without spectators (e.g., FTA, FT%, TO, 3PaR, fouls, and Poss), contributing to the fading of the HA effect during the pandemic season.

## Data availability statement

The datasets presented in this study can be found in online repositories. The names of the repository/repositories and accession number(s) can be found in the article/supplementary material. Further inquiries can be directed to the corresponding author.

## Author contributions

All authors collaborated in the literature review and producing the figure and tables, wrote the manuscript, contributed to the article, and approved the submitted version.

## Footnotes

1
https://www.euroleaguebasketball.net/euroleague/

## References

[B1] Agnew G. A. Carron A. V. (1994). Crowd effects and the home advantage. *Int. J. Sport Psychol.* 25 53–62. 10.12691/rpbs-2-1-1

[B2] Alonso E. Lorenzo A. Ribas C. Gomez M. A. (2022). Impact of COVID-19 pandemic on home advantage in different european professional basketball leagues. *Percept. Mot. Skills* 129 328–342. 10.1177/00315125211072483 35084259 PMC8894916

[B3] Bisciotti G. N. Eirale C. Corsini A. Baudot C. Saillant G. Chalabi H. (2020). Return to football training and competition after lockdown caused by the COVID-19 pandemic: Medical recommendations. *Biol. Sport* 37 313–319. 10.5114/biolsport.2020.96652 32879554 PMC7433324

[B4] Bosquet L. Berryman N. Dupuy O. Mekary S. Arvisais D. Bherer L. (2013). Effect of training cessation on muscular performance: A meta-analysis. *Scand. J. Med. Sci. Sports* 23 e140–e149. 10.1111/sms.12047 23347054

[B5] Bourdas D. I. Mitrousis I. Zacharakis E. D. Travlos A. K. (2022). Home-audience advantage in basketball: Evidence from a natural experiment in EuroLeague games during the 2019-2021 COVID-19 era. *J. Phys. Educ. Sport* 22 1553–1563.

[B6] Bourdas D. I. Zacharakis E. D. Travlos A. K. Souglis A. (2021). Return to basketball play following COVID-19 lockdown. *Sports (Basel)* 9:81. 10.3390/sports9060081 34204988 PMC8228181

[B7] Cao S. Geok S. K. Roslan S. Sun H. Lam S. K. Qian S. (2022). Mental fatigue and basketball performance: A systematic review. *Front. Psychol.* 12:819081. 10.3389/fpsyg.2021.819081 35082736 PMC8784842

[B8] Cohen J. (1992). Statistical power analysis. *Curr. Dir. Psychol. Sci.* 1, 98–101. 10.1111/1467-8721.ep10768783

[B9] Csataljay G. James N. Hughes M. Dancs H. (2012). Performance differences between winning and losing basketball teams during close, balanced and unbalanced quarters. *J. Hum. Sport Exerc.* 7 356–364. 10.4100/jhse.2012.72.02

[B10] Csataljay G. O’donoghue P. Hughes M. Dancs H. (2009). Performance indicators that distinguish winning and losing teams in basketball. *Int. J. Perform. Anal. Sport* 9 60–66. 10.1080/24748668.2009.11868464

[B11] Ehrlich J. Potter J. (2022). Estimating the effect of attendance on home advantage in the National Basketball Association. *Appl. Econ. Lett.* 1–12. 10.1080/13504851.2022.2061898

[B12] Euroleague (2021). *2021-22 euroleague basketball health & safety protocols v1.0.* Barcelona: Euroleague.

[B13] Figueira B. Mateus N. Esteves P. Dadeliene R. Paulauskas R. (2022). Physiological responses and technical-tactical performance of youth basketball players: A brief comparison between 3x3 and 5x5 basketball. *J. Sports Sci. Med.* 21 332–340. 10.52082/jssm.2022.332 35719227 PMC9157511

[B14] Fischer K. Haucap J. (2020). “Betting market efficiency in the presence of unfamiliar shocks: The case of ghost games during the COVID-19 pandemic,” in *Working Paper No. 8526*, (Düsseldorf: Heinrich Heine University Düsseldorf).

[B15] Gomez M. A. Pollard R. (2011). Reduced home advantage for basketball teams from capital cities in Europe. *Eur. J. Sport Sci.* 11 143–148.

[B16] Gomez M. Lorenzo A. Sampaio J. Ibanez S. Ortega E. (2008). Game-related statistics that discriminated winning and losing teams from the Spanish men’s professional basketball teams. *Coll. Antropol.* 32 451–456. 18756894

[B17] Gonçalves B. Coutinho D. Folgado H. Ric A. Malarranha J. Jaime S. (2021). “Strategy, tactics and home advantage in team sports,” in *Home advantage in sport - causes and the effect on performance*, eds Gómez M. Pollard R. Lago-Peñas C. (London: Routledge).

[B18] Harris A. R. Roebber P. J. (2019). NBA team home advantage: Identifying key factors using an artificial neural network. *PLoS One* 14:e0220630. 10.1371/journal.pone.0220630 31365592 PMC6668839

[B19] Harrison A. G. Lin T. Wang P. (2020). Mechanisms of SARS-CoV-2 transmission and pathogenesis. *Trends Immunol.* 41 1100–1115. 10.1016/j.it.2020.10.004 33132005 PMC7556779

[B20] Higgs N. Stavness I. (2021). Bayesian analysis of home advantage in North American professional sports before and during COVID-19. *Sci. Rep.* 11:14521. 10.1038/s41598-021-93533-w 34267238 PMC8282683

[B21] Kozar B. Vaughn R. E. Whitfield K. E. Lord R. H. Dye B. (1994). Importance of free-throws at various stages of basketball games. *Percept. Mot. Skills* 78 243–248. 10.2466/pms.1994.78.1.243

[B22] Kubatko J. Oliver D. Pelton K. Rosenbaum D. T. (2007). A starting point for analyzing basketball statistics. *J. Quant. Anal. Sports* 3 1–24.

[B23] Kunrath C. A. Nakamura F. Y. Roca A. Tessitore A. Teoldo Da Costa I. (2020). How does mental fatigue affect soccer performance during small-sided games? A cognitive, tactical and physical approach. *J. Sports Sci.* 38 1818–1828. 10.1080/02640414.2020.1756681 32362188

[B24] Leota J. Hoffman D. Mascaro L. Czeisler M. Nash K. Drummond S. (2021). Home is where the hustle is: The influence of crowds on effort and home advantage in the National Basketball Association. *SSRN J.* 10.1080/02640414.2022.2154933 36512468

[B25] Link D. Anzer G. (2022). How the COVID-19 pandemic has changed the game of soccer. *Int. J. Sports Med.* 43 83–93. 10.1055/a-1518-7778 34344042 PMC8723889

[B26] Lorenzo Calvo J. Granado-Peinado M. De La Rubia A. Muriarte D. Lorenzo A. Mon-López D. (2021). Psychological States and Training Habits during the COVID-19 Pandemic Lockdown in Spanish Basketball Athletes. *Int. J. Environ. Res. Public Health* 18:9025. 10.3390/ijerph18179025 34501619 PMC8430994

[B27] Lorenzo A. Gómez M. Ortega E. Ibáñez S. J. Sampaio J. (2010). Game related statistics which discriminate between winning and losing under-16 male basketball games. *J. Sports Sci. Med.* 9 664–668. 24149794 PMC3761811

[B28] Mancha-Triguero D. Garcia-Rubio J. Calleja-Gonzalez J. Ibanez S. J. (2019). Physical fitness in basketball players: A systematic review. *J. Sports Med. Phys. Fitness* 59 1513–1525. 10.23736/S0022-4707.19.09180-1 31610639

[B29] Mandic R. Jakovljevic S. Erculj F. Strumbelj E. (2019). Trends in NBA and Euroleague basketball: Analysis and comparison of statistical data from 2000 to 2017. *PLoS One* 14:e0223524. 10.1371/journal.pone.0223524 31589636 PMC6779240

[B30] Mateus N. Gonçalves B. Weldon A. Sampaio J. (2019). Effects of using four baskets during simulated youth basketball games. *PLoS One* 14:e0221773. 10.1371/journal.pone.0221773 31442292 PMC6707597

[B31] Matos R. Amaro N. Pollard R. (2020). How best to quantify home advantage in team sports: An investigation involving male senior handball leagues in Portugal and Spain. *RICYDE. Rev. Int. Cienc. Deport.* 59 12–23. 10.5232/ricyde2020.05902

[B32] McGarry T. (2009). Applied and theoretical perspectives of performance analysis in sport: Scientific issues and challenges. *Int. J. Perform. Anal. Sport* 9 128–140. 10.1186/s12913-016-1423-5 27409075 PMC4943498

[B33] McHill A. W. Chinoy E. D. (2020). Utilizing the National Basketball Association’s COVID-19 restart “bubble” to uncover the impact of travel and circadian disruption on athletic performance. *Sci. Rep.* 10:21827. 10.1038/s41598-020-78901-2 33311539 PMC7732833

[B34] Mikolajec K. Banys D. Zurowska-Cegielska J. Zawartka M. Gryko K. (2021). How to win the basketball euroleague? Game performance determining sports results during 2003-2016 matches. *J. Hum. Kinet.* 77 287–296. 10.2478/hukin-2021-0050 34168711 PMC8008298

[B35] Moore J. C. Brylinsky J. A. (1993). Spectator effect on team performance in college basketball. *J. Sport Behav.* 16 77–84.

[B36] Nevill A. M. Holder R. L. (1999). Home advantage in sport: An overview of studies on the advantage of playing at home. *Sports Med.* 28 221–236. 10.2165/00007256-199928040-00001 10565549

[B37] Oliver D. (2004). *Basketball on paper: Rules and tools for performance analysis.* Sterling, VA: Brassey’s Incorporated.

[B38] Paulauskas R. Masiulis N. Vaquera A. Figueira B. Sampaio J. (2018). Basketball game-related statistics that discriminate between European players competing in the NBA and in the Euroleague. *J. Hum. Kinet.* 65 225–233. 10.2478/hukin-2018-0030 30687434 PMC6341956

[B39] Pojskić H. ŠEparovicì V. UžIčAnin E. (2011). Modelling home advantage in basketball at different levels of competition. *Acta Kinesiol.* 5 25–30.

[B40] Pollard R. Gomez M. A. (2007). Home advantage analysis in different basketball leagues according to team ability. *Eur. J. Sport Sci.* 4 61–64.

[B41] Pollard R. Gomez M. A. (2013). Variations in home advantage in the national basketball leagues of europe. *Rev. Psicol. Del Deporte* 22 263–266.

[B42] Price M. Yan J. (2021). *The Effects of the NBA COVID Bubble on the NBA Playoffs : A Case Study for Home-Court Advantage.* Mansfield: UCONN Library.

[B43] Ric A. Hristovski R. Gonçalves B. Torres L. Sampaio J. Torrents C. (2016). Timescales for exploratory tactical behaviour in football small-sided games. *J. Sports Sci.* 34 1723–1730. 10.1080/02640414.2015.1136068 26758958

[B44] Rodriguez-Fernandez A. Sanchez-Sanchez J. Ramirez-Campillo R. Rodriguez-Marroyo J. A. Vicente J. G. V. Nakamura F. Y. (2018). Effects of short-term in-season break detraining on repeated-sprint ability and intermittent endurance according to initial performance of soccer player. *PLoS One* 13:e0201111. 10.1371/journal.pone.0201111 30110374 PMC6093601

[B45] Salminen S. (1993). The effect of the audience on the home advantage. *Percept. Mot. Skills* 76 1123–1128. 10.2466/pms.1993.76.3c.1123

[B46] Sampaio J. Mcgarry T. Calleja-Gonzalez J. Saiz S. J. Del Alcazar X. S. I. Balciunas M. (2015). Exploring game performance in the national basketball association using player tracking data. *PLoS One* 10:e0132894. 10.1371/journal.pone.0132894 26171606 PMC4501835

[B47] Sors F. Grassi M. Agostini T. Murgia M. (2022). A complete season with attendance restrictions confirms the relevant contribution of spectators to home advantage and referee bias in association football. *PeerJ* 10:e13681. 10.7717/peerj.13681 35811811 PMC9261922

[B48] Sousa A. C. Neiva H. P. Izquierdo M. Cadore E. L. Alves A. R. Marinho D. A. (2019). Concurrent training and detraining: Brief review on the effect of exercise intensities. *Int. J. Sports Med.* 40 747–755. 10.1055/a-0975-9471 31476783

[B49] Tilp M. Thaller S. (2020). COVID-19 has turned home advantage into home disadvantage in the german soccer Bundesliga. *Front. Sports Act. Living* 2:593499. 10.3389/fspor.2020.593499 33345171 PMC7739793

[B50] Unkelbach C. Memmert D. (2010). Crowd noise as a cue in referee decisions contributes to the home advantage. *J. Sport Exerc. Psychol.* 32 483–498. 20733209 10.1123/jsep.32.4.483

[B51] Waters A. Lovelll G. (2002). An examination of the homefield advantage in a professional English soccer team from a psychological standpoint. *Football Stud.* 5 46–59.

[B52] Wu Y. Zeng Y. Zhang L. Wang S. Wang D. Tan X. (2013). The role of visual perception in action anticipation in basketball athletes. *Neuroscience* 237 29–41. 10.1016/j.neuroscience.2013.01.048 23384606

[B53] Wunderlich F. Weigelt M. Rein R. Memmert D. (2021). How does spectator presence affect football? Home advantage remains in European top-class football matches played without spectators during the COVID-19 pandemic. *PLoS One* 16:e0248590. 10.1371/journal.pone.0248590 33788870 PMC8011816

[B54] Ziv G. Lidor R. (2009). Physical attributes, physiological characteristics, on-court performances and nutritional strategies of female and male basketball players. *Sports Med.* 39 547–568. 10.2165/00007256-200939070-00003 19530751

